# Identification of Metabolites of 6′-Hydroxy-3,4,5,2′,4′-pentamethoxychalcone in Rats by a Combination of Ultra-High-Performance Liquid Chromatography with Linear Ion Trap-Orbitrap Mass Spectrometry Based on Multiple Data Processing Techniques

**DOI:** 10.3390/molecules21101266

**Published:** 2016-09-22

**Authors:** Siyi Liu, Yanyun Che, Fei Wang, Zhanpeng Shang, Jianqiu Lu, Shengyun Dai, Jiayu Zhang, Wei Cai

**Affiliations:** 1 School of Chinese Pharmacy, Beijing University of Chinese Medicine, 11 East Road of North 3rd Ring, Chaoyang District, Beijing 100029, China; date641@126.com (S.L.); 18810282932wf@sina.com (F.W.); 13581967977@163.com (Z.S.); lujq@vip.sina.com (J.L.); daisy_1228@126.com (S.D.); 2 School of Chinese Pharmacy, Yunnan University of Traditional Chinese Medicine, Kunming 650500, China; checpu@163.com; 3 Department of Pharmacy, Hunan University of Medicine, Huaihua 41800, China

**Keywords:** 6′-hydroxy-3,4,5,2′,4′-pentamethoxychalcone (PTC), UHPLC-LTQ-Orbitrap MS, metabolite identification, multiple data processing method

## Abstract

In this study, an efficient strategy was established using ultra-high-performance liquid chromatography coupled with linear ion trap-Orbitrap mass spectrometry (UHPLC-LTQ-Orbitrap MS) to profile the in vivo metabolic fate of 6′-hydroxy-3,4,5,2′,4′-pentamethoxychalcone (PTC) in rat urine and feces. The UHPLC-LTQ-Orbitrap method combines the high trapping capacity and MS^n^ scanning function of the linear ion trap along with accurate mass measurements within 5 ppm and a resolving power of up to 30,000 over a wider dynamic range compared to many other mass spectrometers. In order to reduce the potential interferences of endogenous substances, the post-acquisition processing method including high-resolution extracted ion chromatogram (HREIC) and multiple mass defect filters (MMDF) were developed for metabolite detection. As a result, a total of 60 and 35 metabolites were detected in the urine and feces, respectively. The corresponding in vivo reactions such as methylation, hydroxylation, hydrogenation, decarbonylation, demethylation, dehydration, methylation, demethoxylation, sulfate conjugation, glucuronide conjugation, and their composite reactions were all detected in this study. The result on PTC metabolites significantly expanded the understanding of its pharmacological effects, and could be targets for future studies on the important chemical constituents from herbal medicines.

## 1. Introduction

*Murraya paniculata* (L.) Jack (Qianlixiang in Chinese) is widely used in traditional Chinese medicine (TCM). Phytochemical investigations have revealed that flavonoids, coumarins and essential oils are the main constituents of *M. paniculata* [1,2,3]. Polymethoxylated flavonoids (PMFs), a special group of flavonoids, possess a number of interesting biological properties such as anti-allergic, anti-oxidant, anti-bacterial, anti-proliferative and anti-inflammatory activities [4,5]. For example, polymethoxylated chalcones exhibited greater potential antioxidant and anti-inflammatory effects. 6′-Hydroxy-3,4,5,2′,4′-pentamethoxychalcone (PTC) isolated from *M. paniculata* is considered to be the major active compound owing to its special structure and activity [6]. To better understand its mechanism of action and promote its availability as well, the study of its in vivo metabolism behavior is of great importance.

In metabolism studies, it is very important to identify and characterize the chemical structure of metabolites [7,8]. Recently, with the development of various data acquisition methods, liquid chromatography-mass spectrometry (LC-MS), especially with high-resolution mass spectrometry (HRMS), has exhibited excellent performance for metabolite detection because of its high-speed and high detection sensitivity [9,10]. For example, the linear ion trap-Orbitrap mass spectrometer (LTQ-Orbitrap) used here combines a high trapping capacity and the MS^n^ scanning function of the linear ion trap along with accurate mass measurements below 5 ppm and a resolving power of up to 30,000 [11,12,13]. Therefore, it is possible to obtain complex and information—rich metabolic fingerprints in a few minutes—from which *m*/*z* signals of interest could be extracted manually in targeted approaches, or by using statistical tools for global approaches [14,15]. However, many minor metabolites from the full-scan mass chromatograms obtained using LC-MS are likely to be overwhelmed by interferences from the background or the matrix. To detect as many metabolites as possible, off-line data processing such as high-resolution extracted ion chromatogram (HREIC), mass defect filters (MDF) and multiple mass defect filters (MMDF) are also important tecniques for the identification of metabolites [16,17].

In this paper, a comprehensive profiling and identification of in vivo metabolites of PTC in rats was performed. A metabolite identification strategy based on the integration of ultra-high-performance liquid chromatography (UHPLC)-LTQ-Orbitrap MS with multiple data processing techniques was developed for characterizing the major-to-trace metabolites in rat urine and feces. A combination of post-acquisition data-mining methods including HREIC and MMDF were adopted to identify the common and uncommon PTC metabolites. We expect this research will provide a reliable scientific reference and technical guidance for the development and quality evaluation of active components of *M. paniculata*.

## 2. Results

### 2.1. Fragmentation Pathway of Reference Standards

In order to facilitate the structural identification of the metabolites, the MS^n^ fragmentation pattern of four standards were analyzed by UHPLC-LTQ-Orbitrap MS. First, their [M − H]^−^ ions could lose one or more methyl radicals (CH_3_^•^) in the ESI-MS^2^ spectrum, and form [M − H − 15]^−^, [M − H − 30]^−^ and [M − H − 45]^−^ base peaks. Second, the other dissociation pathways of [M − H]^−^ ions by loss of 16 (CH_4_), 28 (CO), 29 (HCO^•^), 31 (OCH_3_^•^), 33 (H_2_O + CH_3_^•^), 43 (CH_3_^•^ + CO), 44 (HCO^•^ + CH_3_^•^), 46 (H_2_O + CO) and 61 (H_2_O + CO + CH_3_^•^) were detected in their MS^n^ spectrum [6]. Meanwhile, a retro-Diels-Alder (RDA) fragmentation reaction cleavage was also observed in the MS spectrum of most compounds, which was useful for PTC metabolite identification.

### 2.2. Establishment of HREIC and MMDF Approach

In order to reduce the potential interferences of endogenous substances, the post-acquisition processing methods HREIC and MMDF was developed for metabolite detection including low levels of predicted and unpredicted metabolites. The HREIC process with calculated *m*/*z* values is highly effective in the detection of common compounds with predictive molecular weights. Meanwhile, MMDF method was implemented using the Metworks software (Thermo Fisher Scientific, Pleasanton, CA, USA) with MDF function for metabolite detection of low levels of unpredicted metabolites, especially the uncommon compounds not detected by HREIC method [13].

Setting the MDF templates is a key step of the MMDF method. The frequently-used templates are: (1) drug filter; (2) substructure filter and (3) conjugate filter. On the basis of the structure of PTC, the following MDF templates were designed: (1) the parent dug (*m*/*z* 373.1281); (2) the demethoxylation product of the drug (*m*/*z* 343.1176); and (3) the combination of glucuronide conjugation (*m*/*z* 549.1602) and sulfate conjugation of PTC (*m*/*z* 453.0849). Specifically, the MDF windows were set to 50 mDa around the mass defect of templates over a mass range of 50 Da of the filter templates [18,19].

### 2.3. Metabolites Identification

**Figure 1:**
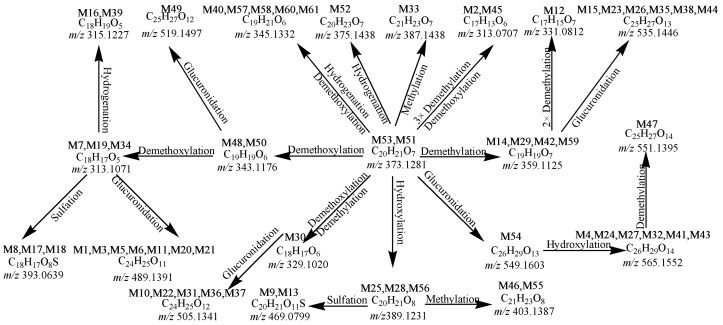
The proposed major metabolic pathway of PTC in the rat urine and feces.

Thermo Xcalibur 2.1 and the Metworks software were used to analyze the data of samples and corresponding blank samples. A total of 60 metabolites of PTC were detected and identified in the urine, and 35 metabolites in the feces using UHPLC-LTQ-Orbitrap mass spectrometry in combination with multiple data mining methods (Table 1 and Figure S1). The proposed major metabolic pathways of PTC in rat urine and feces are illustrated in Figure 1.

**Figure 2:**
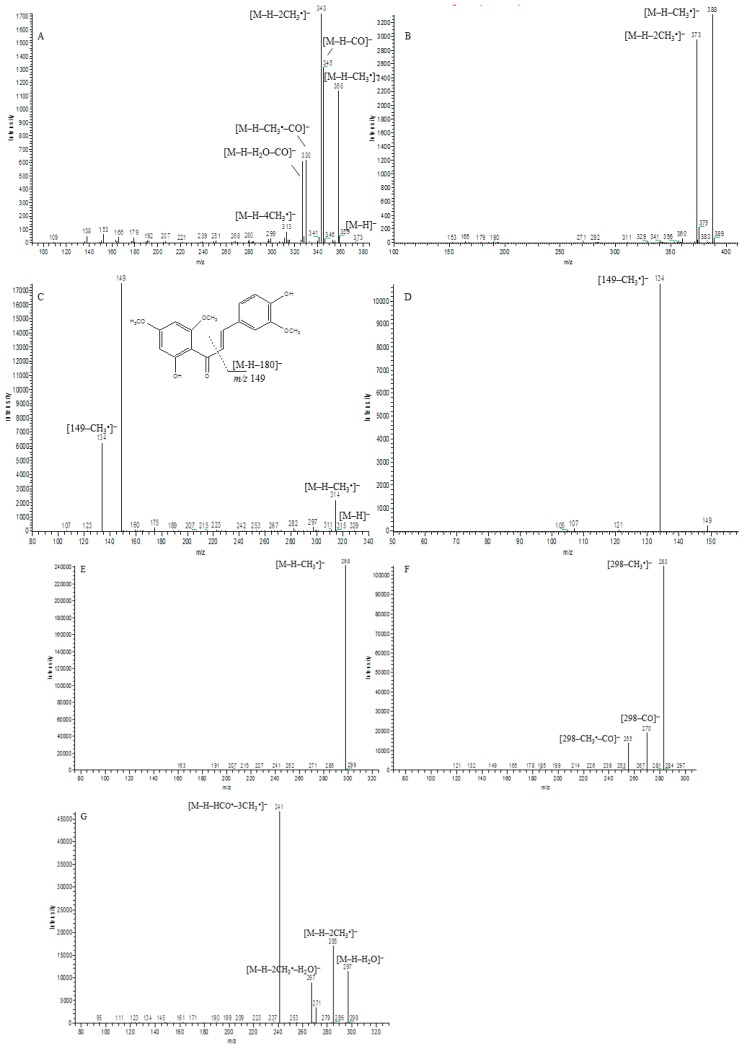
ESI-MS^n^ spectra of four reference standards and **M16**: (**A**) MS^2^ spectrum of PTC; (**B**) MS^2^ spectrum of 6′-hydroxy-3,4,5,2′,3′,4′-hexamethoxychalcone; (**C**) MS^2^ spectrum of 4,6′-dihydroxy-3,2′,4′-trimythoxychalcone; (**D**) MS^3^ spectrum of 4,6′-dihydroxy-3,2′,4′-trimethoxychalcone (precursor-ion was *m/z* 149); (**E**) MS^2^ spectrum of velutin; (**F**) MS^3^ spectrum of velutin (precursor-ion was *m/z* 298); (**G**) MS^2^ spectrum of **M16**.

A total of 60 metabolites were identified and interpreted according to accurate mass measurement, diagnostic fragment ions, relevant drug biotransformation knowledge, and bibliography data. Among them, 60 and 35 metabolites were detected in the urine and feces, respectively. For a better understanding of MS/MS product ions of the metabolites, Mass Frontier 7.0 software and manual elucidation were utilized to propose the fragmentation behaviors of four reference standards in negative-ion mode (Figure 2).

By comparing with the reference standards **M2**, **M30**, **M45**, **M46**, **M51**, **M53** and **M55** were confirmed as velutin, 4,6′-dihydroxy-3,2′,4′-trimethoxychalcone, isomers of velutin, 6′-hydroxy-3,4,5,2′,3′,4′-hexamethoxychalcone, isomers of PTC, prototype and isomers of 6′-hydroxy-3,4,5,2′,3′,4′-hexamethoxychalcone, respectively.

In general, the metabolism of PTC in vivo can be concluded to undergo two pathways, including 17 primary reaction metabolites, and 42 composite reactions metabolites after using three MDF filter templates were detected and identified based on accurate mass measurements, the fragmentation patterns and chromatographic retention times. However, owing to the deficiencies of corresponding reference standards, their structures were tentatively assigned to some extent coupled with Clog*P* values analysis.

The primary reaction metabolites are demethylated (**M14**, **M29**, **M42**, **M59**), methylated (**M33**), demethoxylated (**M48** and **M50**), hydrogenated (**M52**), hydroxylated (**M25**, **M28**, **M56**), and glucuronidated (**M54**) ones. For example, **M14**, **M29**, **M42**, **M59** were eluted at 16.02, 17.93, 19.03 and 26.08 min, possess a deprotonated molecular ion [M − H]^−^ at *m*/*z* 359.11 (C_19_H_19_O_7_), which is 14 Da less than that of PTC, suggesting that they were formed by loss a methyl group from PTC. Meanwhile, the ESI-MS^2^ spectrum of *m*/*z* 359 presented major product ions at *m*/*z* 344 ([M − H − CH_3_^•^]^−^), and gave a prominent [M − H − H_2_O]^−^ at *m*/*z* 341, which are consistent with **M53**. The ESI-MS^2^ spectrum of *m*/*z* 359 gave the [M − H − C_9_H_8_O_4_]^−^ at *m*/*z* 179, suggesting that a RDA reaction had occurred. Therefore, **M14**, **M29**, **M42**, **M59** were tentatively identified as demethylation products of PTC. The Clog*P* value of **M14** is the lowest, suggesting it was the first eluted from a reversed-phase column. Thus, it was tentatively assigned as 2′,4-dihydroxy-3,4′,5,6′-tetramethoxychalcone in combination with Clog*P* analysis. The other three candidates were likewise tentatively characterized. Similarly, metabolite **M54** eluted at 21.60 min, and possesses a deprotonated molecular ion [M − H]^−^ at *m*/*z* 549.1597 (−1.09 ppm, C_26_H_29_O_13_, Clog*P* 0.033), which was 176 Da more than that of **M53**, showing the presence of a glucuronide group. In the MS^2^ spectrum, there was an abundant product ion at *m*/*z* 373 formed by the neutral loss of 176 Da, indicating that the metabolite was a glucuronide conjugate. In the MS^3^ spectrum, the precursor ion at *m*/*z* 373 gave the prominent [M − H − OCH_3_]^−^ at *m*/*z* 343 which was similar to that of **M53**. Therefore, **M54** was tentatively identified as a glucuronide conjugation product of PTC. Finally, on the basis of the Clog*P* value, **M54** were eventually identified.

The second pathway is the composite reactions of the first pathway. For example, metabolites **M16** and **M39** with [M − H]^−^ ions at *m*/*z* 315.1230 (0.82 ppm, C_18_H_19_O_5_, Clog*P* 3.312) and *m*/*z* 315.1229 (0.60 ppm, C_18_H_19_O_5_, Clog*P* 3.742) were eluted at 16.40 min and 18.77 min, respectively. The metabolites have 58 Da less than **M53**, which suggested they were formed by loss of two methoxy groups of PTC and then two hydrogen atoms were added to PTC. By a CID process, [M − H − HCO^•^ − 3CH_3_^•^]^−^ at *m*/*z* 241, [M − H − H_2_O]^−^ at *m*/*z* 297, [M − H − HCO^•^ − CH_3_^•^]^−^ at *m*/*z* 271, [M − H − 2CH_3_^•^]^−^ at *m*/*z* 285 and [M − H − 2CH_3_^•^ − H_2_O]^−^ at *m*/*z* 267 were displayed in their MS^2^ spectrum. Therefore, **M16** and **M39** were tentatively identified as didemethoxylation and hydrogenation product of PTC. According to the value of Clog*P*, **M16** and **M39** were plausibly characterized as 1-(2-hydroxyphenyl)-3-(3,4,5-trimethoxyphenyl)-1-propanone, and 3-(3,4-dimethoxyphenyl)-1-(2-hydroxy-4-methoxy- phenyl)-1-propanone, respectively. The proposed fragmentation behaviors of **M16** in negative ion mode are shown in Figure 2G. Similarly, metabolites **M1**, **M3**, **M5**, **M6**, **M11**, **M20** and **M21** were detected at 12.36, 14.56, 15.10, 15.24, 15.36, 17.02 and 17.25 min, respectively. All of them generated a deprotonated molecule ion at *m*/*z* 489.14 (C_24_H_25_O_11_), or 116 Da more than that of **M53**, suggesting they may be glucuronides, and they were formed by loss of two methoxy groups from PTC. Meanwhile, the ESI-MS^2^ spectrum of *m*/*z* 489 presented major product ions at *m*/*z* 313 ([M − H − C_6_H_8_O_6_]^−^), proving the presence of a glucuronide. By the CID process, [M − H − H_2_O]^−^ at *m*/*z* 471 was displayed in their MS^2^ spectrum, and the ions at *m*/*z* 175 suggested that a RDA reaction had occurred, and further loss of a molecule of H_2_O. Therefore, metabolites **M1**, **M3**, **M5**, **M6**, **M11**, **M20** and **M21** were tentatively identified as didemethoxylation and glucuronidation products of PTC. The Clog*P* value of **M21** is the lowest, suggesting it was difficult for it to be eluted on a reversed-phase column. Thus, it was tentatively assigned as 1-(2-glucuronide-4-methoxyphenyl)-3-(3,5-dimethoxyphenyl)-2-propen-1-one. The other six candidates were likewise tentatively characterized.

## 3. Discussion

Biotransformations of drugs proceed in at least two distinct steps. During first step, compounds are functionalized by demethoxylation, decarbonylation, demethylation, hydrogenation, methylation, hydroxylation, and glucuronidation (primary metabolites). Our results indicated that the α position of PTC was the major metabolic site. In the second step, primary metabolites undergo conjugation reactions or composite reactions to form secondary metabolites. In our study, a possible metabolism pathway for the biotransformation of PTC is established. It is worthwhile to further study the pharmacokinetics and tissue distribution of PTC and its major metabolites [20]. Besides, the introduction of Clog*P* analysis increased the efficiency and accuracy to characterize sequential constituents, especially positional and geometrical isomers.

## 4. Materials and Methods

### 4.1. Chemicals and Materials

**Figure 3:**
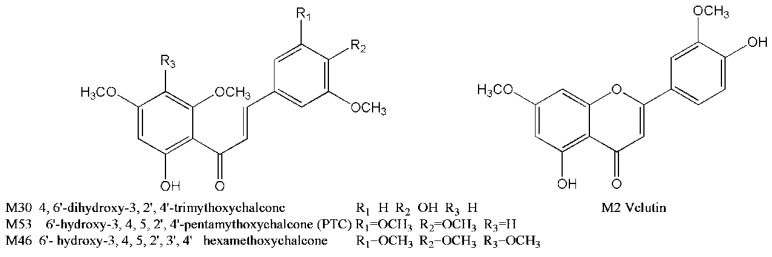
Chemical structures of the reference standards.

Four reference standards including 4,6′-dihydroxy-3,2′,4′-trimethoxychalcone, velutin, 6′-hydroxy-3,4,5,2′,3′,4′-hexamethoxychalcone and 6′-hydroxy-3,4,5,2′,4′-pentamethoxychalcone (PTC) were obtained from the Modern Research Center of Traditional Chinese Medicines, Peking University (Beijing, China) (Figure 3). All these reference compounds showed purities of no less than 95% by HPLC-DAD analysis. Acetonitrile (Fisher, Fair Lawn, NJ, USA), methanol (Fisher, Fair Lawn, NJ, USA) and formic acid (J.T. Baker, Phillipsburg, NJ, USA) were of HPLC grade. Ultra-pure water was prepared by a Milli-Q water purification system (Millipore, Billerica, MA, USA). All the other chemicals of analytical grade were commercially available. *M. paniculata* material was purchased from China Resources Sanjiu Medical & Pharmaceutical Co., Ltd. (Shenzhen, China). It was authenticated by Dr. Peng Tan according to the methods of the Chinese Pharmacopoeia, and its voucher specimen was deposited at the Research Institute of Chinese Medicine, Beijing University of Chinese Medicine.

### 4.2. Animal

Eight Male Sprague-Dawley rats (250 ± 20 g) were obtained from Beijing Weitong Lihua Experimental Animals Company (Beijing, China) and housed at standard temperature (24 ± 2 °C) and humidity (70% ± 5%) in a controlled room with lighting between 07:00 and 19:00 for a week of acclimation. According to the requirements of the National Act on the Use of Experimental Animal (Beijing, China), the animal experiment protocols were approved by the Animal Ethics Committee of Beijing University of Chinese Medicine. After fasting for 12 h with free access to water prior to experiments, rats were randomly divided into two groups (*n* = 8): Group A, drug group for urine and feces, *n* = 4; Group B, control group for blank urine and feces, *n* = 4. All animals had free access to food and water throughout the study.

### 4.3. Drug Administration and Biological Samples Preparation

PTC was suspended in 3% carboxymethyl cellulose sodium (CMC-Na) aqueous solution, and orally administered to rats of Group A at a dose of 50 mg/kg body weight. A 2 mL aliquot of 3% CMC-Na aqueous solution was administrated to each rat in Group B. Urine and feces samples were collected over 0–24 h after gavage. Finally, all biological samples from the same group were merged into one sample. All samples were stored at −20 °C before additional pretreatment.

All the biological samples were prepared by a solid-phase extraction (SPE) method. A SPE cartridge was successively pretreated with methanol and water (5 mL of each), then a 1 mL sample of urine and feces solution was loaded, and allowed to flow through the SPE cartridge under gravity, respectively. Then, the SPE cartridge was successively washed with water (5 mL) and methanol (3 mL). The methanol eluate was collected and evaporated to dryness under N_2_ at room temperature. The residue was re-dissolved in 100 µL of acetonitrile/water (5:95, *v*/*v*) and centrifuged at 14,000 rpm for 10 min. A volume of supernatant (2 µL) was injected into UHPLC-ESI-LTQ-Orbitrap MS for analysis.

### 4.4. Instruments and Conditions

A UHPLC-LTQ-Orbitrap instrument (Thermo Electron, Bremen, Germany) was used to analyse the metabolites, and an ACQUITY UHPLC BEH C_18_ column (1.7 μm, 2.1 mm × 50 mm, Agilent Technologies, Karlsruhe, Germany) was used for the separation. A mobile phase consisting of water containing 0.1% formic acid (A) and acetonitrile (B) was applied with the following program: 0 min, 5% B; 2 min, 5% B; 17 min, 35% B; 23 min, 60% B; 30 min, 80% B; 35 min, 80% B; 36 min, 5% B; 41 min, 5% B. The flow rate was at 0.25 mL/min, and the injection volume was 2 μL.

The ion-source parameters were as follows: sheath gas at 25 arb, auxiliary gas at 3 arb, spray voltage at 4 kV, capillary temperature at 320 °C, tube lens at 120 V and capillary voltage at 30 V. Accurate mass analysis was calibrated according to the manufacturer’s guidelines using a standard solution mix of caffeine, sodium dodecyl sulfate, sodium taurocholate, tetrapeptide MRFA acetate salt and Ultramark. The MS data were collected at 100 ≤ *m*/*z* ≤ 1000 in negative ion mode. Two data acquisition methods were used in the experiments. In one method, a high-resolution scan was conducted using the Orbitrap mass analyzer to acquire MS data at a resolution of 30,000 FWHM. A data-dependent MS^n^ scan was used for the analysis of the MS/MS spectrum generated from the most abundant ions of the MS spectrum. In the other method, a high-resolution scan was conducted using the Orbitrap to acquire the MS data at a resolution of 30,000 FWHM, and the LTQ dynode was used for scanning the MS^2^ and MS^3^ spectrum. The dynamic exclusion function was used to reduce repeat scans, and the repeat count was 2. The exclusion duration was 20 s, and the exclusion mass width was 3 *m*/*z*. The collision energy for collision induced dissociation (CID) was adjusted to 35% of maximum.

### 4.5. Data Processing

A Thermo Xcaliber 2.1 workstation was used for the data acquisition and processing. For the computer-based MDF approach, representative structures with predicted mass defect windows were set as filtering templates for screening homologous compounds. In order to obtain as many fragment ions as possible, the peaks detected with intensity over 10,000 were selected for identification. The chemical formulas for all parent and fragment ions of the selected peaks were calculated from the accurate mass using a formula predictor by setting the parameters as follows: C (0–30), H (0–50), O (0–20), S (0–4), N (0–4), Cl (0–4) and ring double bond (RDB) equivalent value [0–15]. Other elements such as P and Br were not considered as they are rarely present in the complex matrix. The maximum mass errors between the measured and the calculated values were <5 ppm. Blank biological samples were used as control to compare with the analyte samples, and they were all processed under the same conditions. The general procedures of our strategy and approach are summarised in the diagram shown in Figure S2.

## 5. Conclusions

Metabolite identification is important during the early stages of drug discovery and development, as the metabolic products may be pharmacologically active or toxic in nature. In this paper, a UHPLC-LTQ-Orbitrap HREIC and MMDF technique was applied to analyze the metabolism of PTC in rats. A total of 60 metabolites as well as prototype and isomers of PTC, were detected and identified based on accurate mass measurements, the corresponding fragmentation patterns, chromatographic retention times and Clog*P* values. Besides, the results provide comprehensive insights and guidance for elucidation of the mechanism of side effects and safety monitoring as well as for rational formulation design in drug delivery systems. In conclusion, this work profiled the metabolites of PTC in rats which are very useful to understand the in vivo metabolic fate, effective forms and pharmacological and toxic actions of PTC.

## Figures and Tables

**Table 1:** Characterization of metabolites in the rat urine and feces after oral administration of PTC.

Peak	*t*_R_	Theoretical Mass *m*/*z*	Experimental Mass *m*/*z*	Error (ppm)	Formula [M − H]^−^	Clog*P*	MS/MS Fragment Ions	Identification/Reactions	Urine	Feces
**M1**	12.36	489.1391	489.1390	−0.20	C_24_H_25_O_11_	−0.016	313 [M − H − C_6_H_8_O_6_]^−^, 471 [M − H − H_2_O]^−^	2-Propen-1-one,1-(2-glucuronide)-3-(3,4,5-trimethoxyphenyl)	√	√
**M2** *	13.05	313.0707	313.0713	1.90	C_17_H_13_O_6_	3.336	298 [M − H − CH_3_^•^]^−^, 283 [M − H − CO]^−^, 270 [M − H − CH_3_^•^ − CO]^−^, 255 [M − H − 2CH_3_^•^ − CO]^−^	Velutin	√	
**M3**	14.56	489.1391	489.1395	0.82	C_24_H_25_O_11_	0.370	313 [M − H − C_6_H_8_O_6_]^−^, 471 [M − H − H_2_O]^−^, 175 [373 − C_9_H_8_O_4_ − H_2_O]^−^	2-Propen-1-one,1-(2-glucuronide-4-methoxyphenyl)-3-(3,4-dimethoxyphenyl)	√	√
**M4**	14.64	565.1552	565.1542	−1.77	C_26_H_29_O_14_	−1.609	389 [M − H − C_6_H_8_O_6_]^−^, 175 [373 − C_9_H_8_O_4_ − H_2_O]^−^, 521 [M − H − HCO^•^ − CH_3_^•^]^−^, 547(14)	2-Propen-1-one,1-(2-glucuronide-5-hydroxyl-4,6-dimethoxyphenyl)-3-(3,4,5-trimethoxyphenyl)	√	√
**M5**	15.10	489.1391	489.1392	0.20	C_24_H_25_O_11_	0.370	313 [M − H − C_6_H_8_O_6_]^−^, 175 [373 − C_9_H_8_O_4_ − H_2_O]^−^, 471 [M − H − H_2_O]^−^	2-Propen-1-one,1-(2-glucuronide-6-methoxyphenyl)-3-(3,4-dimethoxyphenyl)	√	√
**M6**	15.24	489.1391	489.1393	0.41	C_24_H_25_O_11_	0.370	313 [M − H − C_6_H_8_O_6_]^−^, 175 [373 − C_9_H_8_O_4_ − H_2_O]^−^, 471 [M − H − H_2_O]^−^	2-Propen-1-one,1-(2-glucuronide-4-methoxyphenyl)-3-(3,4-dimethoxyphenyl)	√	√
**M7**	15.28	313.1071	313.1074	0.99	C_18_H_17_O_5_	3.258	295 [M − H − H_2_O]^−^, 269 [M − H − HCO^•^ − CH_3_^•^]^−^	2-Propen-1-one, 1-(2-hydroxyphenyl)-3-(3,4,5-trimethoxyphenyl)	√	√
**M8**	15.28	393.0639	393.0638	−0.25	C_18_H_17_O_8_S	0.998	313 [M − H − SO_3_]^−^	2-Propen-1-one, 1-(2-sulfate)-3-(3,4,5-trimethoxyphenyl)	√	√
**M9**	15.34	469.0799	469.0797	−0.43	C_20_H_21_O_11_S	1.095	257 [M − H − C_9_H_8_O_4_ − 2CH_4_]^−^, 437 [M − H − 2CH_4_]^−^, 389 [M − H − SO_3_]^−^	2-Propen-1-one,1-(2-hydroxy-4,6-dimethoxyphenyl-5-sulfate)-3-(3,4,5-trimethoxyphenyl)	√	
**M10**	15.36	505.1341	505.1341	0.00	C_24_H_25_O_12_	−0.357	329 [M − H − C_6_H_8_O_6_]^−^, 175 [373 − C_9_H_8_O_4_ − H_2_O]^−^	5-glucuronide-2-(3,4,5-trimethoxyphenyl)-4-oxo-1-benzopyran	√	
**M11**	15.36	489.1391	489.1395	0.82	C_24_H_25_O_11_	0.586	313 [M − H − C_6_H_8_O_6_]^−^, 175 [373 − C_9_H_8_O_4_ − H_2_O]^−^, 471 [M − H − H_2_O]^−^	2-Propen-1-one,1-(2-glucuronide-4,6-dimethoxyphenyl)-3-(2-methoxyphenyl)	√	√
**M12**	15.44	331.0812	331.0816	1.21	C_17_H_15_O_7_	0.916	313 [M − H − H_2_O − CO]^−^	2-Propen-1-one,3-(4-hydroxy-3,5-dimethoxyphenyl)-1-(2,4,6-trihydroxyphenyl)	√	
**M13**	15.55	469.0799	469.0798	−0.21	C_20_H_21_O_11_S	1.102	257 [M − H − C_9_H_8_O_4_ − 2CH_4_]^−^, 437 [M − H − 2CH_4_]^−^, 389 [M − H − SO_3_]^−^	2-Propen-1-one,1-(2-hydroxy-4,6-dimethoxyphenyl-)-3-(2-sulfate-3,4,5-trimethoxyphenyl)	√	√
**M14**	16.02	359.1125	359.1133	2.09	C_19_H_19_O_7_	1.754	344 [M − H − CH_3_^•^]^−^, 179 [M − H − C_9_H_8_O_4_]^−^	2′,4-Dihydroxy-3,4′,5,6′-tetramethoxychalcone	√	√
**M15**	16.04	535.1446	535.1443	−0.56	C_25_H_27_O_13_	−0.402	359 [M − H − C_6_H_8_O_6_]^−^, 175 [373 − C_9_H_8_O_4_ − H_2_O]^−^	2-Propen-1-one,1-(2-glucuronide-4,6-dimethoxyphenyl)-3-(3-hydroxy-4,5-dimethoxyphenyl)	√	
**M16**	16.40	315.1227	315.1230	0.82	C_18_H_19_O_5_	3.312	241 [M − H − HCO^•^ − 3CH_3_^•^]^−^, 297 [M − H − H_2_O]^−^, 285 [M − H − 2CH_3_^•^]^−^, 267 [M − H − 2CH_3_^•^ − H_2_O]^−^	1-Propanone,1-(2-hydroxyphenyl)-3-(3,4,5-trimethoxyphenyl)	√	√
**M17**	16.42	393.0639	393.0635	−1.02	C_18_H_17_O_8_S	1.384	313 [M − H − SO_3_]^−^	2-Propen-1-one,1-(2-sulfate-4-methoxyphenyl)-3-(3,4-dimethoxyphenyl)	√	√
**M18**	16.79	393.0639	393.0637	−0.51	C_18_H_17_O_8_S	1.734	313 [M − H − SO_3_]^−^	2-Propen-1-one,1-(2-sulfate-4-methoxyphenyl)-3-(3,5-dimethoxyphenyl)	√	√
**M19**	17.02	313.1071	313.1073	0.70	C_18_H_17_O_5_	3.688	295 [M − H − H_2_O]^−^, 269 [M − H − HCO^•^ − CH_3_^•^]^−^	2-Propen-1-one,3-(3,4-dimethoxyphenyl)-1-(2-hydroxy-4-methoxyphenyl)	√	√
**M20**	17.02	489.1391	489.1394	0.61	C_24_H_25_O_11_	0.586	313 [M − H − C_6_H_8_O_6_]^−^, 175 [373 − C_9_H_8_O_4_ − H_2_O]^−^	2-Propen-1-one,1-(2-glucuronide-4,6-dimethoxyphenyl)-3-(3-methoxyphenyl)	√	√
**M21**	17.25	489.1391	489.1390	−0.20	C_24_H_25_O_11_	0.720	313 [M − H − C_6_H_8_O_6_]^−^, 175 [373 − C_9_H_8_O_4_ − H_2_O]^−^, 471 [M − H − H_2_O]^−^	2-Propen-1-one,1-(2-glucuronide-4-methoxyphenyl)-3-(3,5-dimethoxyphenyl)	√	√
**M22**	17.47	505.1341	505.1342	0.20	C_24_H_25_O_12_	−0.044	329 [M − H − C_6_H_8_O_6_]^−^, 175 [373 − C_9_H_8_O_4_ − H_2_O]^−^, [M − H − H_2_O]^−^	5-Glucuronide-7-methoxy-2-(2-hydroxyl-3,4-dimethoxyphenyl)-4-oxo-1-benzopyran	√	
**M23**	17.58	535.1446	535.1445	−0.19	C_25_H_27_O_13_	−0.468	359 [M − H − C_6_H_8_O_6_]^−^, 175 [373 − C_9_H_8_O_4_ − H_2_O]^−^	2-Propen-1-one,1-(2-glucuronide-4,6-dimethoxyphenyl)-3-(3-hydroxy-4,5-dimethoxyphenyl)	√	
**M24**	17.61	565.1552	565.1549	−0.53	C_26_H_29_O_14_	−1.609	389 [M − H − C_6_H_8_O_6_]^−^, 175 [373 − C_9_H_8_O_4_ − H_2_O]^−^, 521 [M − H − HCO^•^ − CH_3_^•^]^−^, 547 [M − H − 18]^−^	2-Propen-1-one,1-(2-glucuronide-3-hydroxyl-4,6-dimethoxyphenyl)-3-(3,4,5-trimethoxyphenyl)	√	
**M25**	17.64	389.1231	389.1230	−0.26	C_20_H_21_O_8_	2.482	371 [M − H − H_2_O]^−^, 359 [M − H − 2OCH_3_]^−^, 374 [M − H − CH_3_^•^]^−^	2-Propen-1-one,1-(2,5-dihydroxy-4,6-dimethoxyphenyl)-3-(3,4,5-trimethoxyphenyl)	√	√
**M26**	17.69	535.1446	535.1447	0.19	C_25_H_27_O_13_	−1.212	359 [M − H − C_6_H_8_O_6_]^−^, 175 [373 − C_9_H_8_O_4_ − H_2_O]^−^, 517 [M − H − H_2_O]^−^	2-Propen-1-one,1-(2-glucuronide-4,6-dimethoxyphenyl)-3-(5-hydroxy-3,4-dimethoxyphenyl)	√	
**M27**	17.72	565.1552	565.1555	0.53	C_26_H_29_O_14_	−1.609	389 [M − H − C_6_H_8_O_6_]^−^, 175 [373 − C_9_H_8_O_4_ − H_2_O]^−^	2-Propen-1-one,1-(2-glucuronide-4,6-dimethoxyphenyl)-3-(2-hydroxyl-3,4,5-trimethoxyphenyl)	√	
**M28**	17.72	389.1231	389.1231	0.00	C_20_H_21_O_8_	2.238	371 [M − H − H_2_O]^−^, 359 [M − H − 2OCH_3_]^−^, 374 [M − H − CH_3_^•^]^−^	2-Propen-1-one,3-(2-hydroxy-3,4,5-trimethoxyphenyl)-1-(2-hydroxy-4,6-dimethoxyphenyl)	√	
**M29**	17.93	359.1125	359.1125	0.00	C_19_H_19_O_7_	2.781	179 [M − H − C_9_H_8_O_4_]^−^, 341 [M − H − H_2_O]^−^, 344 [M − H − CH_3_^•^]^−^	2-Propen-1-one,1-(2,4-dihydroxy-6-methoxyphenyl)-3-(3,4,5-trimethoxyphenyl)	√	√
**M30** *	18.11	329.1020	329.1018	−0.47	C_18_H_17_O_6_	3.186	149 [M − H − C_9_H_8_O_4_]^−^, 134 [M − H − C_9_H_8_O_4_ − CH_3_^•^]^−^, 314 [M − H − CH_3_^•^]^−^	4,6′-Dihydroxy-3,2′,4′-trimythoxychalcone	√	√
**M31**	18.11	505.1341	505.1343	0.39	C_24_H_25_O_12_	−0.044	329 [M − H − C_6_H_8_O_6_]^−^, 175 [373 − C_9_H_8_O_4_ − H_2_O]^−^, [M − H − H_2_O]^−^	5-Glucuronide-7-methoxy-2-(2-hydroxyl-3,4-dimethoxyphenyl)-4-oxo-1-benzopyran	√	
**M32**	18.18	565.1552	565.1554	0.35	C_26_H_29_O_14_	−1.609	389 [M − H − C_6_H_8_O_6_]^−^, 175 [373 − C_9_H_8_O_4_ − H_2_O]^−^, 547 [M − H − 18]^−^	2-Propen-1-one,1-(2-glucuronide-4,6-dimethoxyphenyl)-3-(6-hydroxyl-3,4,5-trimethoxyphenyl)	√	√
**M33**	18.26	387.1438	387.1441	0.77	C_21_H_23_O_7_	3.127	369 [M − H − H_2_O]^−^, 343 [M − H − HCO^•^ − CH_3_^•^]^−^	2′,3,4,4′,5,6′-Hexamethoxychalcone	√	√
**M34**	18.37	313.1071	313.1071	0.31	C_18_H_17_O_5_	4.038	295 [M − H − H_2_O]^−^, 269 [M − H − HCO^•^ − CH_3_^•^]^−^	2-Propen-1-one,3-(3,5-dimethoxyphenyl)-1-(2-hydroxy-4-methoxyphenyl)	√	√
**M35**	18.43	535.1446	535.1448	0.37	C_25_H_27_O_13_	−0.316	359 [M − H − C_6_H_8_O_6_]^−^	2-Propen-1-one,1-(2-glucuronide-4-hydroxy-6-methoxyphenyl)-3-(3,4,5-trimethoxyphenyl)	√	
**M36**	18.46	505.1341	505.1345	0.79	C_24_H_25_O_12_	0.068	329 [M − H − C_6_H_8_O_6_]^−^, 175 [373 − C_9_H_8_O_4_ − H_2_O]^−^, [M − H − H_2_O]^−^	2-Propen-1-one,1-(2-glucuronide-4-hydroxy)-3-(3,4-dimethoxyphenyl)	√	
**M37**	18.54	505.1341	505.1343	0.39	C_24_H_25_O_12_	0.306	329 [M − H − C_6_H_8_O_6_]^−^, 175 [373 − C_9_H_8_O_4_ − H_2_O]^−^, [M − H − H_2_O]^−^	5-glucuronide-7-methoxy-2-(2-hydroxyl-3,5-dimethoxyphenyl)-4-oxo-1-benzopyran	√	
**M38**	18.69	535.1446	535.1449	0.56	C_25_H_27_O_13_	−0.368	359 [M − H − C_6_H_8_O_6_]^−^, 175 [373 − C_9_H_8_O_4_ − H_2_O]^−^, 517 [M − H − H_2_O]^−^	2-Propen-1-one,1-(2-glucuronide-4,6-dimethoxyphenyl)-3-(4-hydroxy-3,5-dimethoxyphenyl)	√	
**M39**	18.77	315.1227	315.1229	0.60	C_18_H_19_O_5_	3.742	297 [M − H − H_2_O]^−^, 271 [M − H − HCO^•^ − CH_3_^•^]^−^	1-Propanone,3-(3,4-dimethoxyphenyl)-1-(2-hydroxy-4-methoxyphenyl)	√	√
**M40**	18.83	345.1332	345.1333	0.29	C_19_H_21_O_6_	3.384	327 [M − H − H_2_O]^−^	1-Propanone,3-(2,3,4-trimethoxyphenyl)-1-(2-hydroxy-4-methoxyphenyl)	√	√
**M41**	18.89	565.1552	565.1550	−0.35	C_26_H_29_O_14_	0.144	389 [M − H − C_6_H_8_O_6_]^−^, 359 [M − H − C_6_H_8_O_6_ − OCH_3_]^−^, 175 [373 − C_9_H_8_O_4_ − H_2_O]^−^	2-Propen-1-one,1-(2-hydroxyl-4-glucuronide-6-methoxyphenyl)-3-(6-hydroxyl-3,4,5-trimethoxyphenyl)	√	
**M42**	19.03	359.1125	359.1129	1.17	C_19_H_19_O_7_	2.869	344 [M − H − CH_3_^•^]^−^, 341 [M − H − H_2_O]^−^, 179 [M − H − C_9_H_8_O_4_]^−^	2-Propen-1-one,2-Propen-1-one,1-(2,6-dihydroxy-4-methoxyphenyl)-3-(3,4,5-trimethoxyphenyl)	√	√
**M43**	19.20	565.1552	565.1553	0.18	C_26_H_29_O_14_	0.144	389 [M − H − C_6_H_8_O_6_]^−^, 359 [M − H − C_6_H_8_O_6_ − OCH_3_]^−^	2-Propen-1-one,1-(2-hydroxyl-4,6-dimethoxyphenyl)-3-(3-glucuronide-4,5-dimethoxyphenyl)	√	
**M44**	19.43	535.1446	535.1447	0.19	C_25_H_27_O_13_	0.144	359 [M − H − C_6_H_8_O_6_]^−^, 175 [373 − C_9_H_8_O_4_ − H_2_O]^−^	2-Propen-1-one, 1-(2-glucuronide-4-methoxyphenyl-6- hydroxyl)-3-(3,4,5-trimethoxyphenyl)	√	
**M45**	19.49	313.0707	313.0707	0.24	C_17_H_13_O_6_	−	298 [M − H − CH_3_^•^]^−^	Isomers of velutin	√	
**M46** *	20.06	403.1387	403.1390	0.74	C_21_H_23_O_8_	2.541	388 [M − H − CH_3_^•^]^−^	6′-Hydroxy-3,4,5,2’,3’,4’-hexamethoxy-chalcone	√	√
**M47**	20.11	551.1395	551.1395	0.00	C_25_H_27_O_14_	−1.224	375 [M − H − C_6_H_8_O_6_]^−^, 533 [M − H − H_2_O]^−^, 175 [373 − C_9_H_8_O_4_ − H_2_O]^−^	5-Glucuronide-7-methoxy-2-(2-hydroxy-3,4,5-trimethoxyphenyl)-4-oxo-1-benzopyran	√	
**M48**	20.20	343.1176	343.1176	0.00	C_19_H_19_O_6_	3.330	315 [M − H − CO]^−^, 313 [M − H − 2CH_3_^•^]^−^, 328 [M − H − CH_3_^•^]^−^, 300 [M − H − CH_3_^•^ − CO]^−^, 297 [M − H − H_2_O − CO]^−^	2-Propen-1-one,1-(2-hydroxy-4-methoxyphenyl)-3-(3,4,5-trimethoxyphenyl)	√	
**M49**	20.63	519.1497	519.1495	−0.38	C_25_H_27_O_12_	0.144	343 [M − H − C_6_H_8_O_6_]^−^, 175 [373 − C_9_H_8_O_4_ − H_2_O]^−^	2-Propen-1-one,1-(2-glucuronide-4-methoxyphenyl-6-hydroxy)-3-(3,4,5-trimethoxyphenyl)	√	√
**M50**	20.67	343.1176	343.1177	0.29	C_19_H_19_O_6_	3.662	315 [M − H − CO]^−^, 313 [M − H − 2CH_3_^•^]^−^, 328 [M − H − CH_3_^•^]^−^, 300 [M − H − CH_3_^•^ − CO]^−^, 297 [M − H − H_2_O − CO]^−^	2-Propen-1-one,3-(3,4-dimethoxyphenyl)-1-(2-hydroxy-4,6-dimethoxyphenyl)	√	√
**M51**	20.71	373.1281	373.1283	0.54	C_20_H_21_O_7_	−	343 [M − H − 2CH_3_^•^]^−^, 345 [M − H − CO]^−^, 358 [M − H − CH_3_^•^]^−^, 330 [M − H − CH_3_^•^ − CO]^−^, 327 [M − H − H_2_O − CO]^−^	Isomers of PTC	√	
**M52**	20.89	375.1438	375.1438	0.00	C_20_H_23_O_7_	3.358	328 [M − H − H_2_O − HCO^•^]^−^, 345 [M − H − CH_3_^•^]^−^, 357 [M − H − H_2_O]^−^	2-Propen-1-one,1-(2-hydroxy-4,6-dimethoxyphenyl)-3-(3,4,5-trimethoxyphenyl)	√	√
**M53** *	21.58	373.1281	373.1279	−0.54	C_20_H_21_O_7_	3.304	343 [M − H − 2CH_3_^•^]^−^, 345 [M − H − CO]^−^, 358 [M − H − CH_3_^•^]^−^, 330 [M − H − CH_3_^•^ − CO]^−^, 327 [M − H − H_2_O − CO]^−^	Prototype	√	
**M54**	21.60	549.1603	549.1597	−1.09	C_26_H_29_O_13_	0.033	373 [M − H − C_6_H_8_O_6_]^−^, 343 [M − H − C_6_H_8_O_6_ − OCH_3_]^−^	2-Propen-1-one,1-(2-glucuronide-4,6-dimethoxyphenyl)-3-(3,4,5-trimethoxyphenyl)	√	√
**M55**	21.86	403.1387	403.1388	0.25	C_21_H_23_O_8_	−	388 [M − H − CH_3_^•^]^−^	Isomers of 6′-hydroxy-3,4,5,2′,3’,4’-hexamethoxychalcone	√	
**M56**	22.06	389.1231	389.1234	0.77	C_20_H_21_O_8_	2.488	371 [M − H − H_2_O]^−^	2-Propen-1-one,1-(1,2-dihydroxy-3,6-dimethoxyphenyl)-3-(3,4,5-trimethoxyphenyl)	√	√
**M57**	22.76	345.1332	345.1335	0.87	C_19_H_21_O_6_	3.384	277 [M − H − 2H_2_O − 2CH_4_]^−^, 327 [M − H − H_2_O]^−^	1-Propanone,3-(2,3,4-trimethoxyphenyl)-1-(2-hydroxy-6-methoxyphenyl)	√	√
**M58**	22.93	345.1332	345.1336	1.16	C_19_H_21_O_6_	3.716	327 [M − H − H_2_O]^−^, 309 [M − H − 2H_2_O]^−^, 277 [M − H − 2H_2_O − 2CH_4_]^−^	1-Propanone,3-(3,4-dimethoxyphenyl)-1-(2-hydroxy-4,6-dimethoxyphenyl)	√	
**M59**	26.08	359.1125	359.1130	1.39	C_19_H_19_O_7_	2.869	344 [M − H − CH_3_^•^]^−^	2-Propen-1-one,1-(2-hydroxy-4,6-dimethoxyphenyl)-3-(2-hydroxy-3,4-dimethoxyphenyl)	√	√
**M60**	29.31	345.1332	345.1337	1.45	C_19_H_21_O_6_	3.716	277 [M − H − 2H_2_O − 2CH_4_]^−^	1-Propanone,3-(3,4-dimethoxyphenyl)-1-(2-hydroxy-4,6-dimethoxyphenyl)	√	√
**M61**	31.07	345.1332	345.1335	0.87	C_19_H_21_O_6_	4.066	277 [M − H − 2H_2_O − 2CH_4_]^−^	β-(2,4-dimethoxyphenyl)-2-hydroxy-4,6-dimethoxypropiophenone	√	√

* Compared with a reference compound.

## Supplementary Materials

Supplementary materials can be accessed at: http://www.mdpi.com/1420-3049/21/10/1266/s1.
