# Supplementary material for: Identification of Metabolites of 6′-Hydroxy-3,4,5,2′,4′-pentamethoxychalcone in Rats by a Combination of Ultra-High-Performance Liquid Chromatography with Linear Ion Trap-Orbitrap Mass Spectrometry Based on Multiple Data Processing Techniques
Source: Molecules. 2016 Sep 22;21(10):1266. doi: 10.3390/molecules21101266 (PMC6272839; doi:10.3390/molecules21101266)
Supplement: Supplementary file 1 [file molecules-21-01266-s001.pdf]

Supplementary Materials: Identification of Metabolites of 6′-Hydroxy-3,4,5,2′,4′-pentamythoxychalcone in Rats by Combination of Ultra–High–Performance Liquid Chromatography with Linear Ion Trap–Orbitrap Mass Spectrometer Based on Multiple Data Processing Techniques

Siyi Liu, Yanyun Che, Fei Wang, Zhanpeng Shang, Jianqiu Lu, Shengyun Dai, Jiayu Zhang and Wei Cai

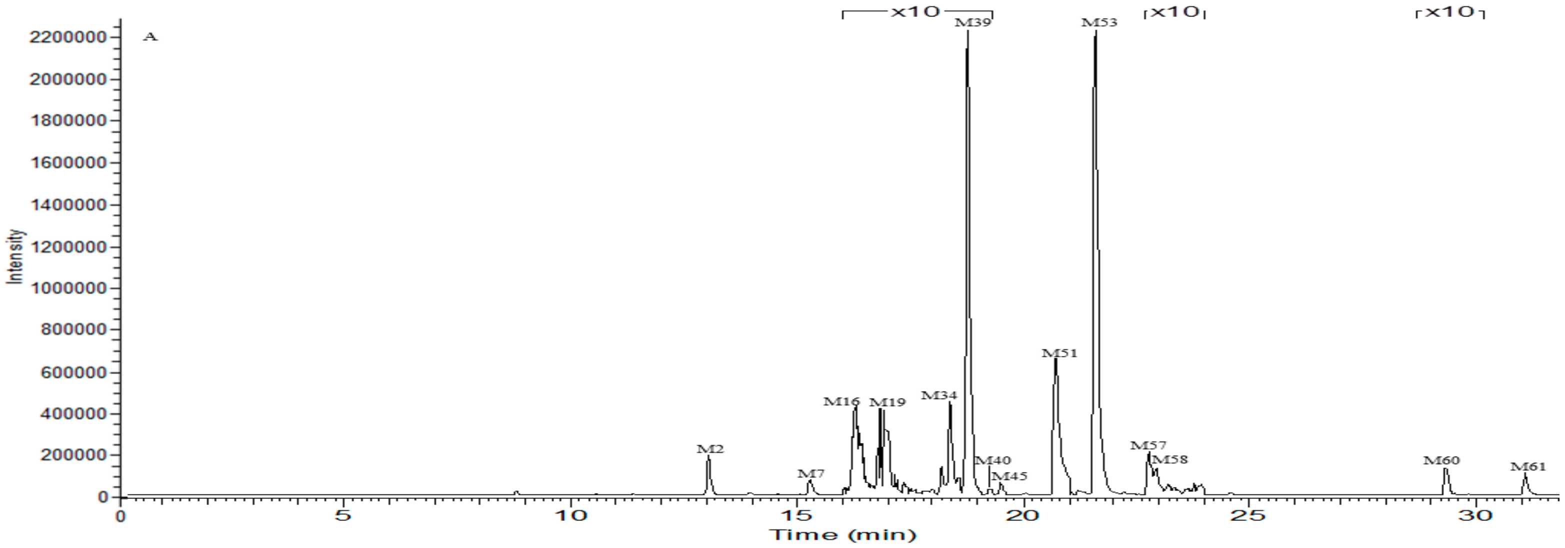

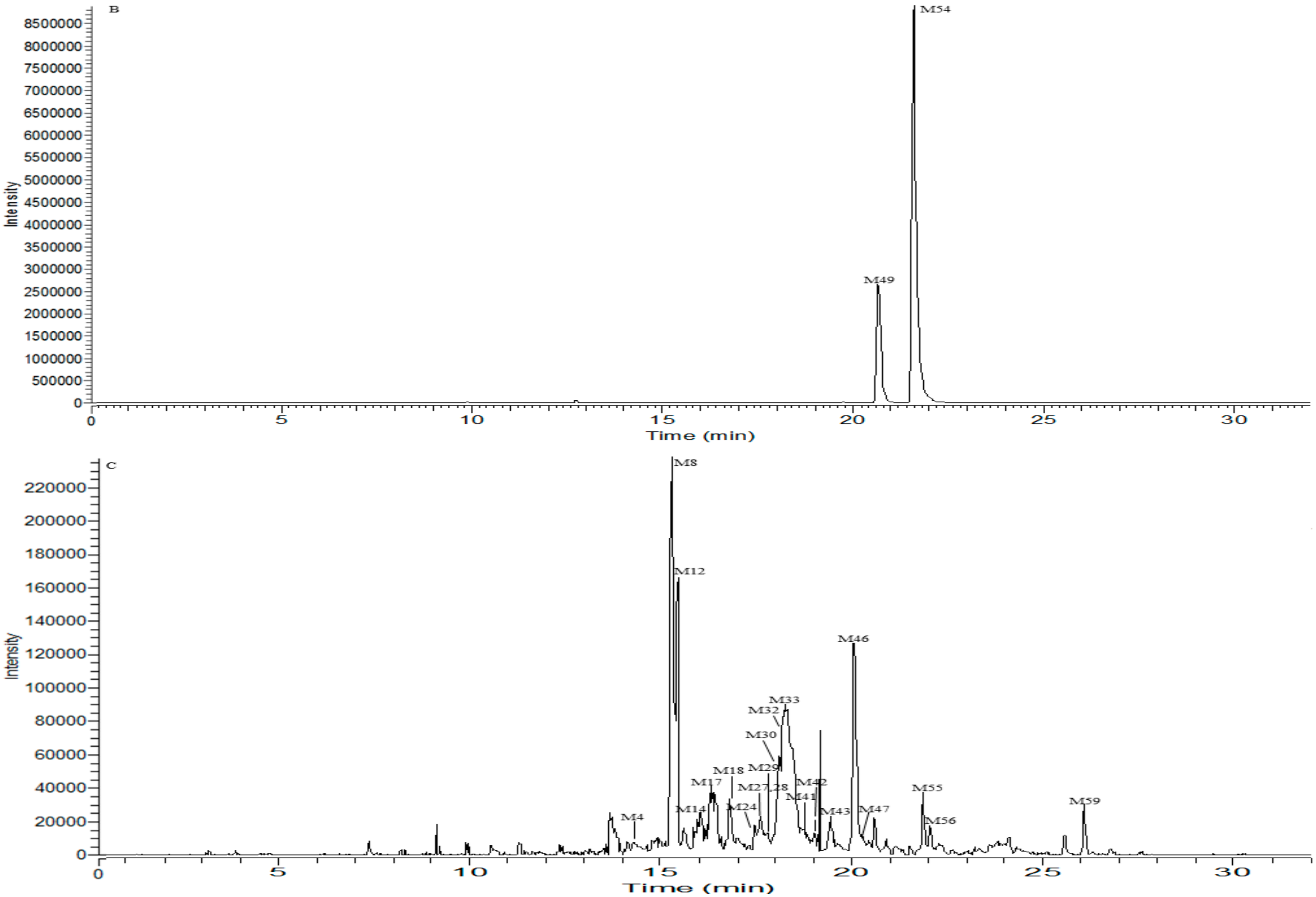
B
M54
M49
Intensity
Time (min)
C
M8
M12
M46
M33
M32
M30
M29
M18
M17
M27,28
M42
M41
M14
M24
M43
M47
M4
M55
M56
M59
Intensity
Time (min)

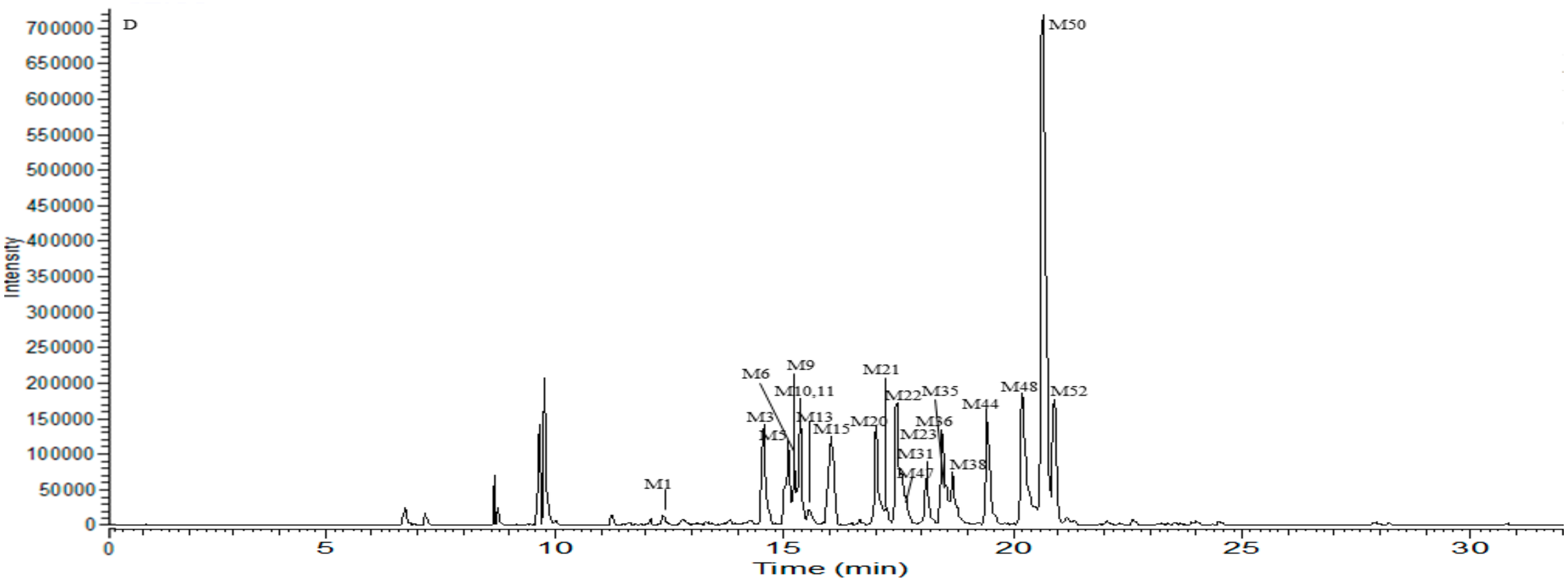

Figure S1. The high-resolution EIC in 5 ppm for the multiple metabolites in rat urine and feces (**A**) *m/z* 373.1281, 313.1071, 315.1227, 313.0707, 345.1332; (**B**) *m/z* 549.1603, 519.1497; (**C**) *m/z* 331.0812, 329.1020, 359.1125, 387.1438, 389.1231, 393.0639, 403.1387, 551.1395, 565.1552; (**D**) *m/z* 343.1176, 469.0799, 505.1341, 535.1446, 489.1391, 375.1438.

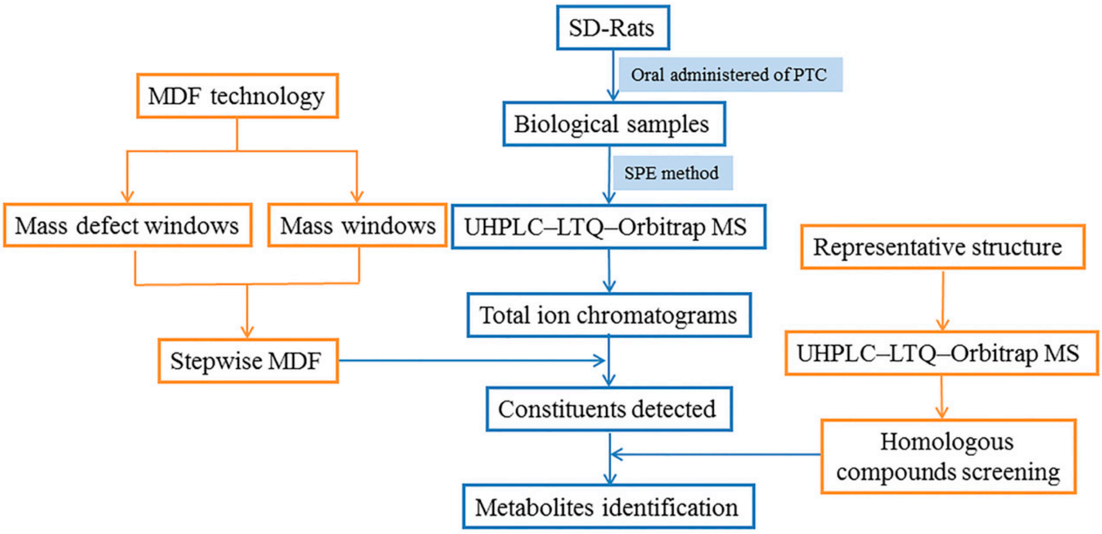

Figure S2. Summary diagram of the developed strategy and methodology.
